# Uncovering Community Structures with Initialized Bayesian Nonnegative Matrix Factorization

**DOI:** 10.1371/journal.pone.0107884

**Published:** 2014-09-30

**Authors:** Xianchao Tang, Tao Xu, Xia Feng, Guoqing Yang

**Affiliations:** 1 School of Computer Science and Technology, Tianjin University, Tianjin, China; 2 School of Computer Science and Technology, Civil Aviation University of China, Tianjin, China; 3 Information Technology Research Base of Civil Aviation Administration of China, Tianjin, China; Semmelweis University, Hungary

## Abstract

Uncovering community structures is important for understanding networks. Currently, several nonnegative matrix factorization algorithms have been proposed for discovering community structure in complex networks. However, these algorithms exhibit some drawbacks, such as unstable results and inefficient running times. In view of the problems, a novel approach that utilizes an initialized Bayesian nonnegative matrix factorization model for determining community membership is proposed. First, based on singular value decomposition, we obtain simple initialized matrix factorizations from approximate decompositions of the complex network’s adjacency matrix. Then, within a few iterations, the final matrix factorizations are achieved by the Bayesian nonnegative matrix factorization method with the initialized matrix factorizations. Thus, the network’s community structure can be determined by judging the classification of nodes with a final matrix factor. Experimental results show that the proposed method is highly accurate and offers competitive performance to that of the state-of-the-art methods even though it is not designed for the purpose of modularity maximization.

## Introduction

Many complex systems in the real world have the form of networks whose edges are linked by nodes or vertices. Examples include social systems such as personal relationships, collaborative networks of scientists, and networks that model the spread of epidemics; ecosystems such as neuron networks, genetic regulatory networks, and protein-protein interactions; and technology systems such as telephone networks, the Internet and the World Wide Web [1]. In these networks, there are many sub-graphs, called communities or modules, which have a high density of internal links. In contrast, the links between these sub-graphs have a fairly lower density [2]. In community networks, sub-graphs have their own functions and social roles. Furthermore, a community can be thought of as a general description of the whole network to gain more facile visualization and a better understanding of the complex systems. In some cases, a community can reveal the real world network’s properties without releasing the group membership or compromising the members’ privacy. Therefore, community detection has become a fundamental and important research topic in complex networks.

**Figure 1:**
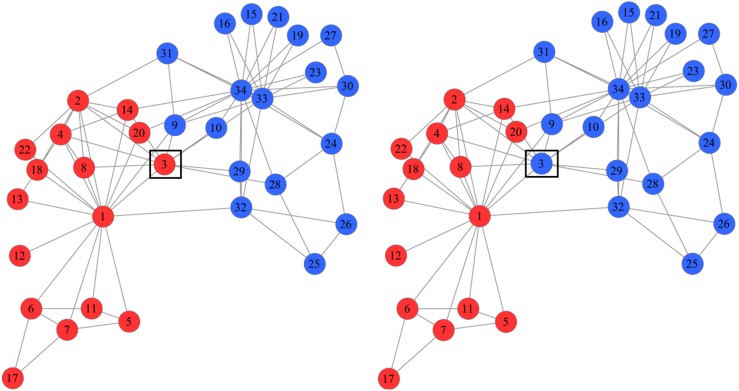
A comparison of BNMF with two random initializations.

In recent decades, a number of methods have been developed for community detection in which an objective function is maximized or minimized. One of these community detection methods is nonnegative matrix factorization (NMF), which was proposed by Lee and Seung [3]. Using the matrix factorization method, one can find the community membership of each vertex in a network. Several improvements of the NMF have been proposed, such as the Bayesian nonnegative matrix factorization (BNMF) approach for identifying overlapping communities, which was presented by Psorakis et al. [4]; the symmetric nonnegative matrix factorization (SNMF) technique for detecting overlapping communities proposed by Wang et al. [5]; and the bounded NMF (BNMTF) technique for community detection proposed by Zhang and Yeung [6]. NMF is a nonconvex optimization problem with the inequality constraints shown in Eq. (1), and iterative methods are required to obtain the solution.
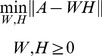
(1)However, the current NMF methods converge slowly and at local minima [7]. Most of the algorithms in the literature randomly initialize 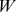 and 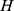. The results of these algorithms are not unique when using different initializations, such as those obtained using BNMF to detect a karate network, which is shown in Figure 1. Therefore, several instances are needed to obtain a better solution; however, this process is expensive.

Several methods have been adapted for initializing NMF. For example, Meyer et al. [8] use the *“random Acol”* method, which takes the average of 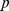 random rows as the initialization for NMF. Wild et al. [9] use *“Clustering Centroid”*, which uses the centroid vector for initialization. Another important initialization method is NNDSVD (nonnegative double singular value decomposition), which was proposed by C. Boutsidis and E. Gallopoulos [7]. NNDSVD uses the rank-2 matrix with the nearest positive approximation as its initialization and obtains better results than other initialization methods.

In this paper, we present a novel and running time efficient method for community detection based on BNMF with a simple NNDSVD approximation as the initialization, which we call IBNMF, to determine the community membership. The merits of this approach are as follows: 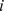) computationally efficient and stable, 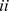) high accuracy in determining the membership of networks, and 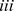) overcoming the drawbacks of the maximum modularity criterion.

## Methods

In this section, we introduce the community discovery framework of our method. Then, we test the performance of our approach on a range of synthetic networks and real-world benchmark examples and provide experimental evidence of the effectiveness of the proposed algorithm.

### Community Discovery Framework of IBNMF

**Figure 2:**
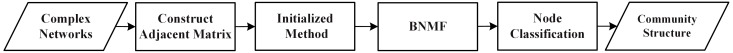
The community discovery framework of IBNMF.

Our community discovery framework for complex networks is shown in Figure 2. First, we construct the networks’ adjacency matrix from the original data. Then, using the simple NNDSVD method, the initialization of 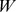 and 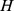 can be obtained. Thereafter, we combine the initialized 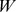 and 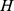 and BNMF to acquire the final matrix factor 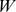 after several iterations. Lastly, the matrix factor is used to determine the community membership.

#### Adjacency matrix

For a given non-weighted undirected network 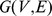 whose vertex set is 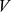 and whose edge set is 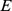, we use an adjacency matrix 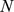 to describe the network. When nodes 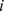 and 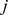 are connected by an edge, the element 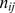 is set to 1; otherwise, this element is set to 0. The diagonal elements are usually 0, but by considering the difference between the zero elements on the diagonal and off-diagonal, we set the degree of node 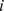 as the value for each diagonal element 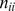.

#### Node classification

For an 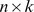 factor matrix 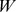, 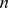 is the number of total nodes in the network, 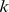 is the number of the sub-networks in the social network, and the element 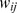 represents the probability of the 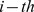 node being in the 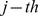 community. In this work, we select the principle of probability maximization to determine the community to which the node belongs: if 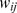 is the largest element in the 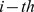 row 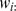, then node 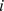 is part of community 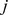.

In the following section, we give the theoretical foundations of the singular value decomposition (SVD) initialization method and the IBNMF algorithm.

### Simple NNDSVD Initialization

The SVD [10] of an 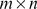 matrix 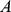 involves the factorization of 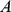 into three matrices 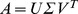, where both 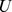 and 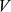 are orthogonal matrices, and 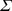 is an diagonal matrix with following form:
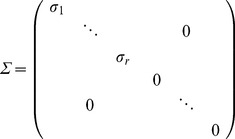
(2)In the above matrix, 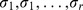 are the singular values of *A*. For each 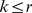, the rank-*k* approximation of the matrix *A* based on Frobenius norm can be written as [7]:
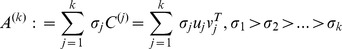
(3)In the F-norm, each 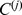 can be best approximated by the nonnegative section 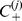. We use the modification shown in expansion (3) to produce the nonnegative approximation of *A* and to obtain effective initial values for *W* and *H* to determine the community membership.

To reduce the running time, the following two steps are used in this paper to obtain a quick approximation of the network’s adjacency matrix: first, the maximum rank of 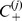 is set to 1. We use the main component 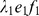 of 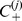 as an approximation of 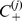 because this component contains most of the information in the networks. Secondly, because 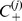 is the nearest positive approximation of 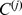, we can use 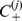 as the approximation of 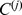. Hence, if *A = U
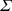

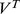
* is the decomposition of *A* by SVD, then we have 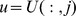 and 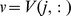. We initialize the column and row vectors in *W* and *H,* respectively, using the equations below.
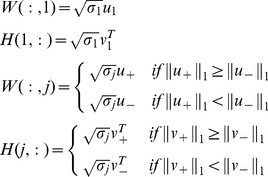
(4)From the preceding results, it is possible to approximate the factors (*W*, *H*) as follows: *i*) perform a SVD of *A* with descending eigenvalues, *ii*) compute the first column and row vectors in *W* and *H* with Eq.(4), *iii*) compute the subsequent column and row vectors in *W* and *H* with Eq. (4), and *iv*) use the results as an initialization of the network’s adjacency matrix.

### Bayesian Nonnegative Matrix Factorization

**Figure 3:**
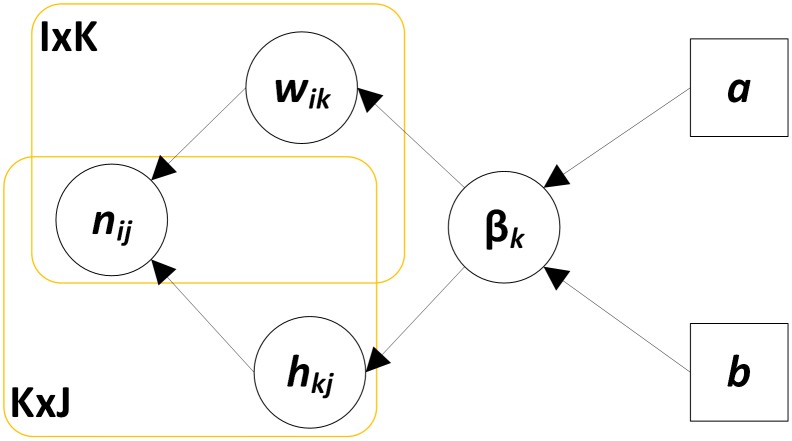
A directed graphical model illustrates BNMF. This graphical model describes the generation of 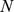 from 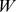 and 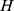 with the components’ scale hyperparameters 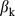; 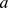 and 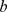 are fixed parameters.

BNMF follows the generative model in Figure 3 [11], where the detected nonnegative value 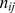 denotes interactions occurring between two nodes *i* and *j* in the network with adjacency matrix 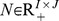. In the process of interactions, two nonnegative matrices 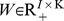 and 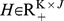 are found such that 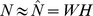. BNMF assumes that each single element 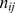 of *N* obeys Poisson distribution at a rate 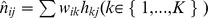. In the nonnegative matrices *W* and *H*, rank *K* is the number of groups or communities in the networks, whose initial value is unknown. By using scale hyperparameters 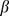 that control the importance of the community in both the columns of *W* and the rows of *H* [12], the values of these hyperparameters and the values of *W* and *H* can be iteratively inferred by maximizing the posterior of the parameters given by the data [13]. To be specific, the precise values of *W*, *H* and 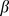 can be obtained by optimizing the maximum a posteriori criterion:
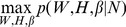
(5)


Maximizing the posterior criterion is equivalent to minimizing a cost function *F* in (6).
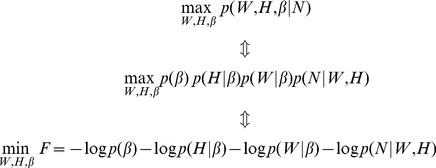
(6)


Considering the priors for *W* and *H* and the parameters’ probability distribution (standard Gamma distribution over 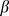 [13], half-normal probability distribution of *W* and *H* parameterized by precision 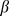 [13]–[17], and Poisson distribution of *N* over 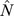 [11], [13]), the optimization model is.
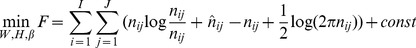
(7)


According to the expression for *F*, the object function can be minimized by optimizing the sum of *W*, *H*, and 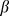
’s log-likelihoods. Considering [2], [13], [18], [19] and adopting the update algorithm proposed in [13], we obtain the update steps in Algorithm 1 with an algorithmic complexity of 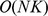.

**Algorithm 1** Community Detection using IBNMF
**Input:**
  Nonnegative matrix 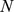, initial 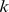, fixed Gamma hyperparameters 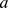, 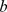;
**Output:**

Nonnegative matrices 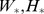;

1: [m, n] = size(*N*); W  =  zeros(m, *k*); H  =  zeros(*k*, n);

2: [U,S,V]  =  psvd (*N*, *k*);
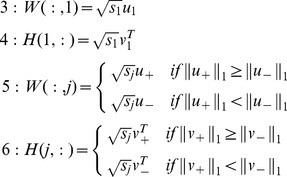



7: **for** each 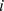 in 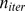 **do**

8: 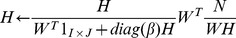


9: 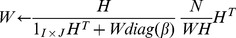


10: 
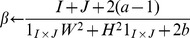


11: check termination criterion: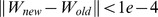;//community structure is stable.

12: **end for**

13: **return** 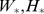


## Results and Discussion

In this section, we used both synthetic (computer-generated) and real-world networks to show IBNMF’s effectiveness. The synthetic datasets enable us to test the algorithm’s performance and stability, and the real datasets allow us to observe the method’s accuracy under practical, real-world conditions.

### Synthetic Networks

**Figure 4:**
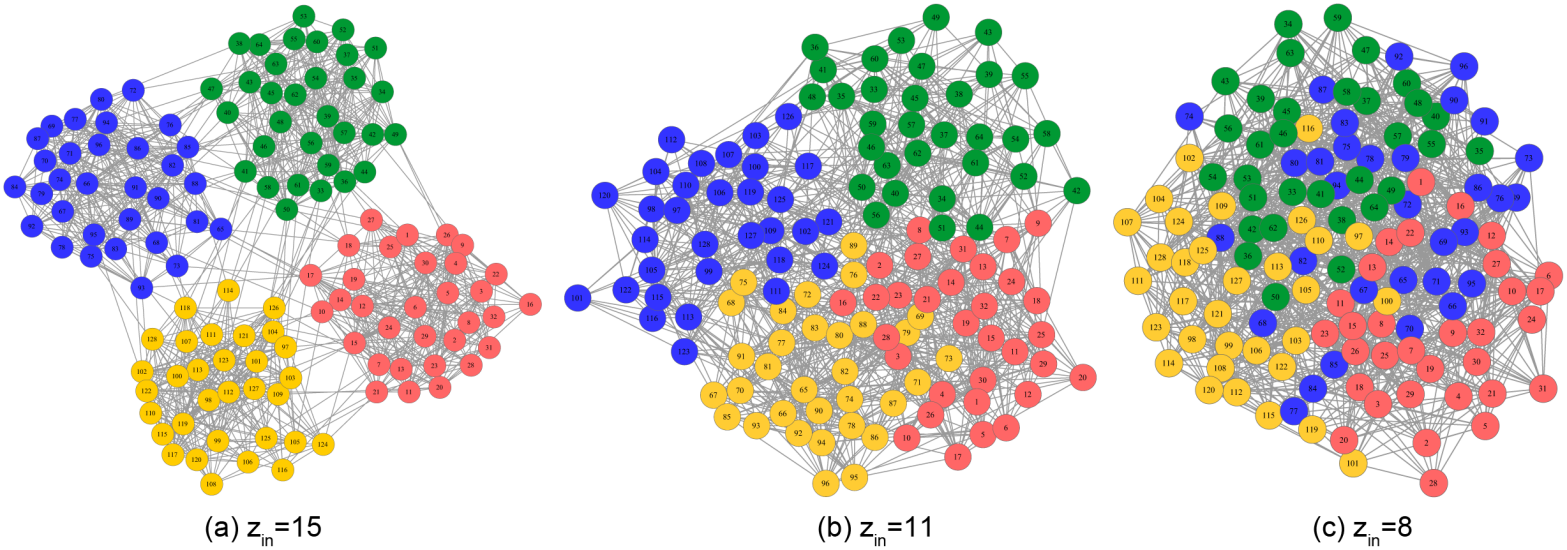
Three different parameter GN benchmark networks. Each graph contains 128 vertices, and each vertex is connected to exactly 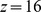 others. These vertices are divided into four separate communities: 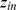 is the number of intra-community connections made to members of its own community whereas 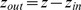 is the number of an inter-community connections with other communities.

Our first synthetic network examples employ Newman’s large set of artificial, computer-generated benchmark networks (GN benchmarks) [1]. Each graph was constructed with 128 vertices, and each vertex was connected to exactly 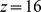 others. These vertices were divided into four separate communities such that some number 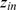 of each vertex’s 16 connections were made to randomly chosen members of its own community while the remaining 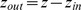 connections were made to random members of other communities. This process produces graphs that have a known community structure, but are essentially random in other respects. As shown in Figure 4, when 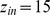, the vertexes have more intra-community connections than inter-community ones; when 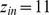, the vertexes also have more intra-community connections than inter-community ones; finally, when 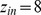, the vertexes have as many intra-community connections as inter-community ones. Note that in the third graph, the community structure is not clear and the vertices cannot be accurately divided into four parts as in the first and second graphs.

To evaluate the performance and stability of IBNMF with respect to determining the community structure, we choose the widely used measure called modularity 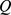 [20], [21], which can be given by:
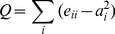
(8)


The modularity is the sum of the sub-modularities in different communities [20], which measures the density of intra-community connections and inter-community connections.

**Figure 5:**
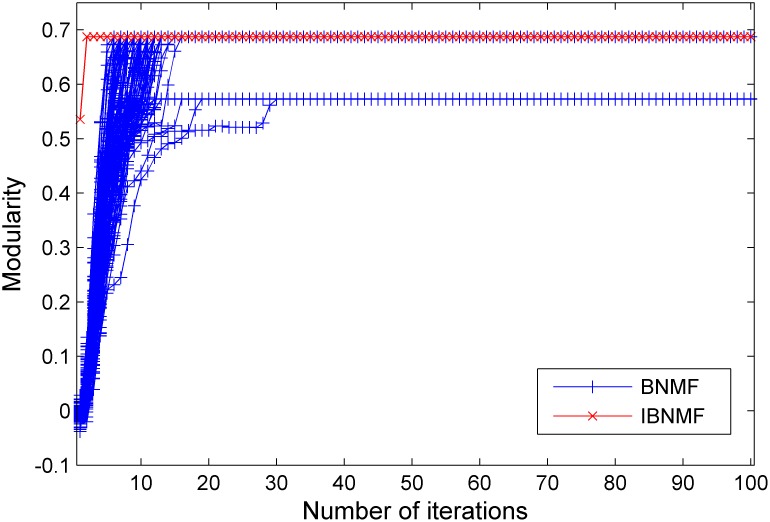
A comparison between our simple NNDSVD initialization method and a random initialization method. The results are given in terms of modularity for a GN benchmark network with 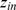 = 15.

**Figure 6:**
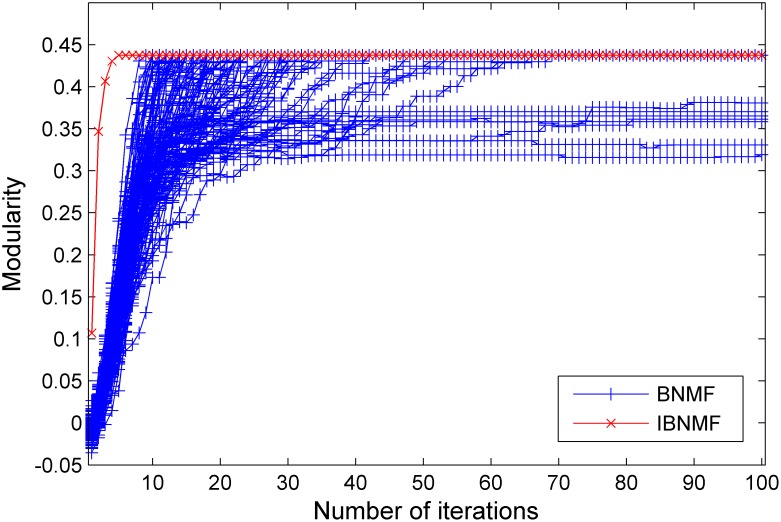
A comparison between our simple NNDSVD initialization method and a random initialization method. The results are given in terms of modularity for a GN benchmark network with 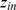 = 11.

**Figure 7:**
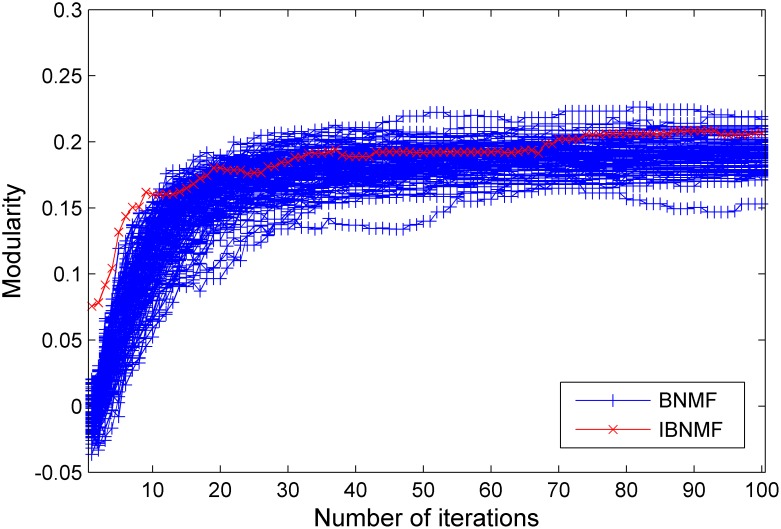
A comparison between our simple NNDSVD initialization method and a random initialization method. The results are given in terms of modularity for a GN benchmark network with 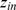 = 8.

Using the synthetic benchmark networks, we tested the modularity and stability of our algorithm in comparison with the random initialization method (BNMF) as the ratio of intra-community connections to inter-community connections varied. After running our method and the random initialization method 100 times, we obtained the 600 sets of results shown in Figures 5, 6 and 7.

In these figures, we give the results of the two algorithms in terms of their stability and average performance as measured by the modularity. Generally, the experimental performance of IBNMF is better than that of the random initialization algorithm in terms of modularity. When 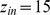 and 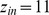, our method has a higher initial modularity and converges more rapidly to a better final result, and the final stable modularity is also higher than that of the random initialization method. Furthermore, when 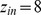, we also obtain a higher initial modularity and an average solution even though the network cannot be appropriately divided. Furthermore, the most important fact is that our method gives a unique solution for 100 experiments, as indicated by the red lines in Figures 5, 6 and 7.

In short, when the community structure is clear, as shown in Figure 4, IBNMF obtains a stable solution that does not change as the number of iterations increases, and this solution is obtained in fewer steps than with BNMF. In addition, when the community structure is not clear, our method produces a unique solution, as represented by the red line, which is better than the BNMF results in terms of the average modularity.

**Figure 8:**
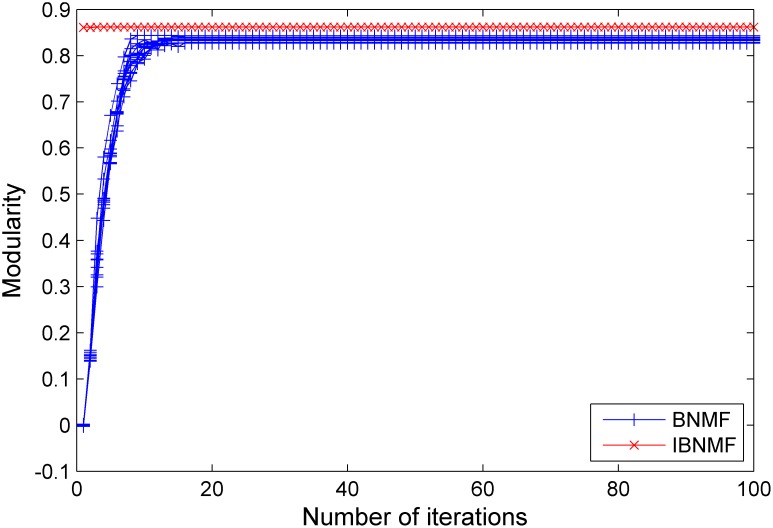
A comparison between our simple NNDSVD initialization method and a random initialization method. The results are given in terms of modularity for an LFR benchmark network with 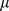 = 0.1.

**Figure 9:**
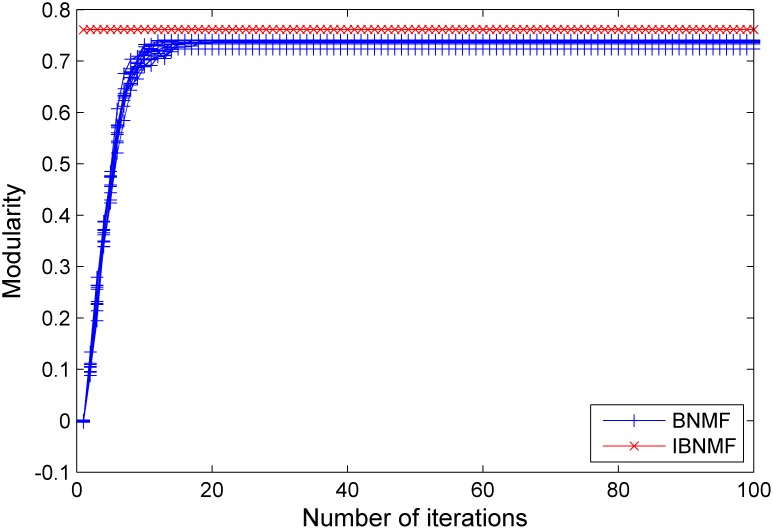
A comparison between our simple NNDSVD initialization method and a random initialization method. The results are given in terms of modularity for an LFR benchmark network with 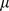 = 0.2.

**Figure 10:**
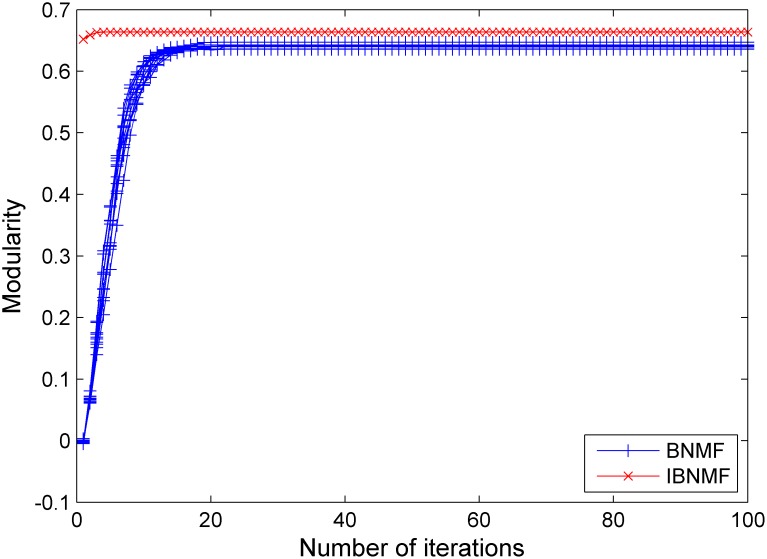
A comparison between our simple NNDSVD initialization method and a random initialization method. The results are given in terms of modularity for an LFR benchmark network with 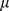 = 0.3.

Our second synthetic network examples are based on a Lancichinetti-Fortunato-Radicchi (LFR) benchmark network [22], which more accurately reflects the properties of real-world networks. In LFR benchmark networks, distributions of node degrees and community sizes follow power laws with exponents 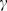 and 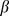. The network cohesion is controlled by two mixing parameters 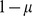 and 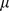, which denote the fraction of a node’s neighbors in its own community and the fraction of neighbors that are in the other communities, respectively. In this paper, the parameters of the LFR benchmark were set as follows: the number of nodes equals 1000, the average degree is 15, the maximum degree is equal to 50, and the mixing parameter 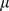 ranges from 0.1 to 0.3. The number of runs is set to 10. Moreover, we evaluate the performance and stability of IBNMF using modularity; the results presented in Figures 8, 9 and 10 demonstrate that our IBNMF method has a higher initial modularity and rapidly converges to a better final result.

### Sensitivity Analysis

**Figure 11:**
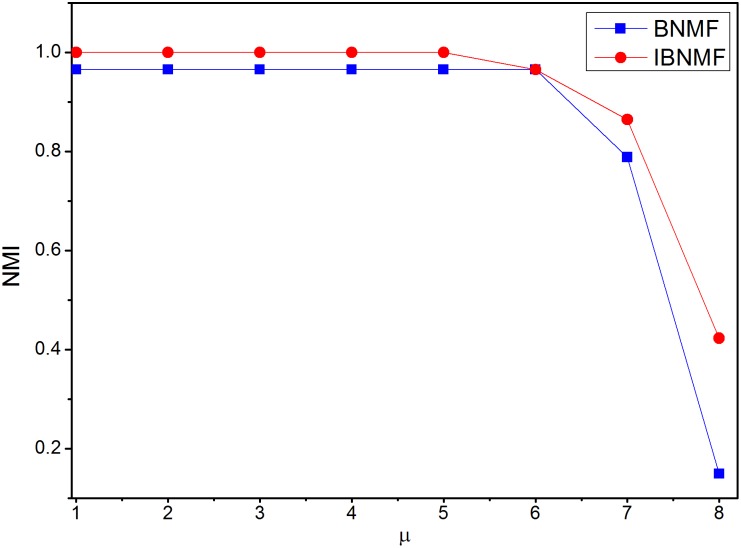
Average normalized mutual information for GN benchmarks.

**Figure 12:**
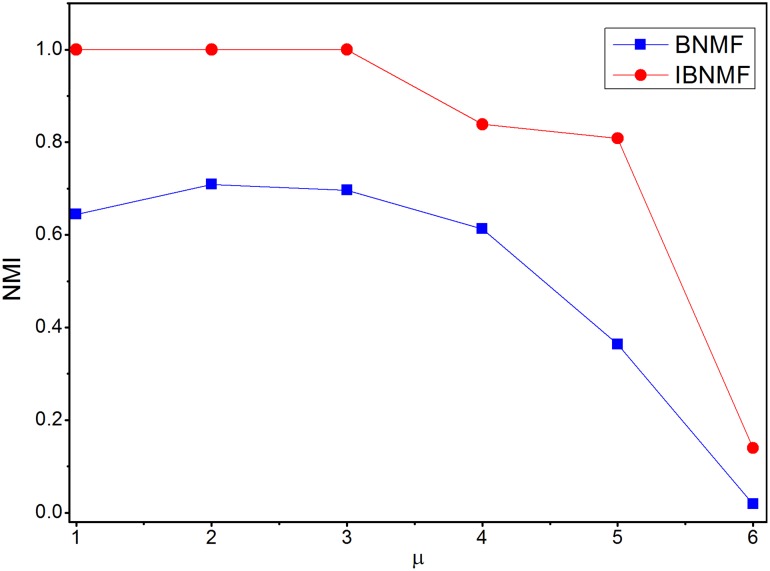
Average normalized mutual information for LFR benchmarks.

Furthermore, we use normalized mutual information (NMI) [23] to evaluate the sensitivity of our method on synthetic networks (GN and LFR). The free parameters used here include 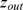 and 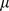. We vary parameter 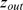 from 1 to 8 and parameter 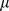 from 0.1 to 0.6. The number of runs is set to 10, and the average NMI results are shown in Figures 11 and 12. From these two figures, one can observe the following: (i) the results of both the BNMF and IBNMF models decrease as 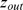 or 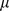 increases; and (ii) IBNMF consistently outperforms BNMF on both benchmarks. From the above results, we can also see that IBNMF outperforms BNMF with respect to the iteration times. The detailed iteration times of IBNMF and BNMF that are required to obtain a steady solution are shown in Tables 1 and 2.

**Table 1:** Iteration times for GN benchmarks.

	GN( 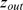 )							
	1	2	3	4	5	6	7	8
IBNMF	3	4	5	3	5	7	19	19
BNMF	9	11	10	11	19	16	30	39

**Table 2:** Iteration times for LFR benchmarks.

	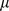					
	0.1	0.2	0.3	0.4	0.5	0.6
IBNMF	1	2	2	10	14	20
BNMF	11	17	21	20	37	40

To analyze the sensitivity of the modularity for different priors, we perform a statistical analysis of the mean and variance by using simple NNDSVD and the random initialization, as shown in Table 3. From the experimental results, one can observe the following: (i) IBNMF obtains a higher mean modularity value than random initialization BNMF; and (ii) the simple NNDSVD initialization model is more stable than the random initialization model. The higher mean value and lower variance indicate that IBNMF has better and more stable performance for the GN and LFR benchmarks.

**Table 3:** Summary of statistics for modularity based on the use of different priors.

	Simple NNDSVD		Random	
	Mean	Variance	Mean	Variance
GN( 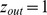 )	0.6716	6.1007e–031	0.6482	0.0023
GN( 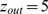 )	0.4418	1.5252e–031	0.4267	8.7437e–004
GN( 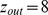 )	0.2347	2.5212e–031	0.2306	2.7471e–004
LFR( 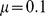 )	0.8617	0	0.8326	3.9899e–005
LFR( 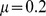 )	0.7616	0	0.7314	2.7275e–005
LFR( 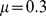 )	0.6636	1.3696e–032	0.6416	1.9708e–005

We have also tested our method on numerous real-world networks. In the next section, we provide detailed accuracy results of our method for the community detection of specific examples.

### Real Networks

While synthetic networks provide a reproducible and well-controlled testing platform for our community structure algorithm, it is desirable to test the algorithm on real-world networks as well. To this end, we selected ten datasets representing real-world communities and compared the results of IBNMF with those of several state-of-the-art methods. In Table 4, our real-world network datasets are described by the vertex number 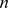, edge number 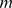 and actual community number 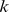. “Friendship6” network and “Friendship7” network are the same high school friendship network based on two different ground-truths [24]. All of the networks that we used here were obtained from Newman’s website [25], except for “Friendship”, which was obtained from Add Health in [26]. The methods that we used for comparison include the Louvain method [27], which is one of the best approaches for vertex partition [24]; Newman’s fast algorithm [28], which is one of the most widely used methods for community detection; the mixed-membership stochastic block model (MMSB) [29], which is based on a Bayesian model of networks that allows nodes to participate in multiple communities; RN [30], which is based on a minimization of the Hamiltonian of a Potts-like spin model; Infomap [31], which is based on optimally compressing the information in the structure of the graph; BNMTF and SNMF methods, which are NMF based community detection ones; and other initialization methods.

**Table 4:** Ten real-world datasets used in this work.

Dataset	*n*	*M*	*K*	Description
Karate	34	78	2	Karate club [32]
Dolphins	62	159	2	Dolphin network [33]
Friendship6	68	220	6	High school friendship [26]
Friendship7	68	220	7	High school friendship [26]
Polbooks	105	441	3	US politics books [34]
Word	112	425	2	Word network [35]
Polblogs	1490	16718	-	Blogs about politics [36]
Football	115	613	-	American college football [21]
Net Science	1589	2742	-	Scientific collaboration networks [37]
Email	1133	5451	-	Email network [38]

To compare the performances of our method with the algorithms mentioned above, we adopt accuracy comparison and community modularity as measures for real-word datasets.

#### Accuracy comparisons

Various measures can be used to compare the given community structure with the one discovered by the algorithm. Here, we take fraction of vertices classified correctly (FVCC) [1], as a metric of accuracy comparison. The methods for comparison include the following: Louvain, RN, Infomap, BNMTF, and SNMF. Newman’s fast algorithm is not included in this comparison, as it was not designed for FVCC. To test the influence of simple NNDSVD and a random initialization method, SNMF, SSNMF, IBNMF, and BNMF are also compared in our experiment. Furthermore, to test the influence of simple NNDSVD and other initialization methods, RCBNMF and CBNMF are also included. The abbreviations of the various initialized NMFs are introduced in Table 5.

**Table 5:** A comparison of IBNMF with the Louvain, BNMTF, BNMF, RCBNMF, CBNMF, SSNMF and RSNMF methods for six real networks with FVCC.

FVCC	IBNMF	Louvain	BNMTF	BNMF	RCBNMF	CBNMF	SSNMF	SNMF
Karate	100	97.10	93.62	79.41	100	95.88	100	91.76
Dolphins	96.77	96.67	82.97	83.39	87.29	73.39	91.94	85.79
Friendship6	84.06	92.70	76.35	88.99	91.16	88.15	84.06	84.19
Friendship7	92.75	91.30	87.58	91.45	90.87	89.30	92.75	86.70
Polbooks	82.86	84.80	72.91	79.60	81.11	78.63	81.90	74.85
Word	66.38	58.95	59.58	57.34	63.71	61.20	54.46	56.10

The abbreviations of different initialized nonnegative matrix factorizations: RCBNMF is BNMF with *“random Acol”* initialization; CBNMF is BNMF with clustering initialization; SSNMF is SNMF with our initialization.

Table 5 and 6 are the experimental results of different community detection algorithms based on FVCC index. As can be seen, IBNMF gives better results than other community detection methods and has the best performance in real-world networks. Compared with the random initialization method, simple NNDSVD initialization gives better results: both BNMF and SNMF have better performance on real-world networks. In addition, compared with other initialization methods such as *“random Acol”* and clustering, simple NNDSVD initialization also gives the best performance. In fact, IBNMF requires fewer iterations to obtain a unique result than the other initialization methods.

**Table 6:** A comparison of IBNMF with MMSB, RN, and Infomap for six real networks with FVCC.

FVCC	IBNMF	MMSB	RN	Infomap
Karate	100	94.12	64.71	82.35
Dolphins	96.77	62.90	98.39	58.06
Friendship6	84.06	84.06	18.84	84.06
Friendship7	92.75	75.36	18.84	92.75
Polbooks	82.86	76.19	80.95	78.10
Word	66.38	55.36	49.11	51.79

#### Modularity comparisons

As mentioned above, modularity is one of the most widely used indexes for community detection. Here, we select the modularity as our second evaluation criterion. In previous experiments, NNDSVD initialization has exhibited better performance than the other initialization methods. Thus, the methods for comparison include the Louvain method, MMSB, RN, Infomap, Newman’s fast algorithm, SSNMF, and BNMTF.

Table 7 gives the results of different algorithms in terms of the average modularity. As can be seen, our IBNMF has competitive performance even though it was not designed for the purpose of modularity maximization, unlike Louvain and Newman’s fast method. Furthermore, our algorithm has the advantage of providing higher accuracy for community detection. In conclusion, our approach gives a better and more stable result than other initialization methods with a shorter running time.

**Table 7:** A comparison of IBNMF with the Louvain, MMSB, RN, Infomap, Newman’s fast, SSNMF and BNMTF methods for nine real networks with modularity.

Dataset	IBNMF	Louvain	MMSB	RN	Infomap	Newman’s fast	SSNMF	BNMTF
Karate	0.406	0.419	0.332	0.406	0.402	0.379	0.388	0.372
Dolphins	0.512	0.514	0.253	0.379	0.529	0.500	0.507	0.507
Friendship	0.586	0.590	0.500	0.400	0.595	0.585	0.583	0.524
Polbooks	0.519	0.520	0.451	0.527	0.527	0.486	0.506	0.492
Word	0.227	0.291	0.121	–0.0002	0.031	0.291	0.284	0.267
Polblogs	0.509	0.425	0.230	…	0.425	0.419	0.413	0.404
Football	0.594	0.604	0.261	0.601	0.601	0.572	0.592	0.570
Net Science	0.821	0.848	0.734	0.734	0.807	0.848	0.804	0.782
Email	0.540	0.548	0.190	0.008	0.538	0.477	0.532	0.511

## Conclusions

In this paper, we present a novel method, IBNMF, for community detection, which adopts a simple NNDSVD initialization based on BNMF to achieve better and more stable results than other community detection methods. Experimental results show that IBNMF can determine the community membership in both synthetic and real-world networks. The proposed approach is more accurate and offers competitive performance to that of the RN, Infomap, Louvain and Newman’s fast methods even though it is not designed for the purpose of modularity maximization. In contrast to other initialized NMF methods, our method is computationally efficient and obtains a better and more stable result with less running time.
