# Correction: Uncovering Community Structures with Initialized Bayesian Nonnegative Matrix Factorization

**DOI:** 10.1371/journal.pone.0115880

**Published:** 2014-12-11

**Authors:** The PLOS ONE Staff

The following information is missing from the Funding section: this work is supported by the National Science & Technology Pillar Program (2014BAJ04B02).
